# Evolutionary Divergence in DNA Damage Responses among Fungi

**DOI:** 10.1128/mBio.03348-20

**Published:** 2021-03-16

**Authors:** Jacob L. Steenwyk

**Affiliations:** a Department of Biological Sciences, Vanderbilt University, Nashville, Tennessee, USA

**Keywords:** cell cycle, DNA damage, DNA repair, budding yeasts, Saccharomycotina

## Abstract

Cell cycle checkpoints and DNA repair pathways contribute to maintaining genome integrity and are thought to be evolutionarily ancient and broadly conserved. For example, in the yeast Saccharomyces cerevisiae and humans, DNA damage induces activation of a checkpoint effector kinase, Rad53p (human homolog Chk2), to promote cell cycle arrest and transcription of DNA repair genes. However, recent studies have revealed variation in the DNA damage response networks of some fungi. For example, Shor et al. (mBio 11:e03044-20, 2020, https://doi.org/10.1128/mBio.03044-20) demonstrate that in comparison to S. cerevisiae, the fungal pathogen Candida glabrata has reduced activation of Rad53p in response to DNA damage. Consequently, some downstream targets that contribute to S. cerevisiae genome maintenance, such as DNA polymerases, are transcriptionally downregulated in C. glabrata. Downregulation of genome maintenance genes likely contributes to higher rates of mitotic failure and cell death in C. glabrata. This and other recent findings highlight evolutionary diversity in eukaryotic DNA damage responses.

## Abstract (precis)

Cell cycle checkpoints and DNA repair pathways contribute to maintaining genome integrity and are thought to be evolutionarily ancient and broadly conserved. For example, in the yeast Saccharomyces cerevisiae and humans, DNA damage induces activation of a checkpoint effector kinase, Rad53p (human homolog Chk2), to promote cell cycle arrest and transcription of DNA repair genes.

## COMMENTARY

Organisms are challenged by a constant barrage of exogenous and endogenous DNA-damaging agents (1). To cope with the threat of potentially deleterious mutations, an intricate network of DNA damage response processes, including cell cycle checkpoints and DNA repair pathways, help detect and repair DNA lesions (2). Due to their fundamental importance to life, most cell cycle and DNA repair processes are thought to be ancient in origin and broadly conserved (3).

One broadly conserved process is phosphorylation-based activation of the checkpoint effector kinase Rad53p (human homolog Chk2) in the presence of DNA damage or replication fork stalling (4–7). Phosphorylation of Rad53p by upstream sensor kinases and autophosphorylation amplifies the DNA damage signal, leading to cell cycle arrest, transcription of DNA repair genes, replication fork stabilization, and the activation of other processes that contribute to genome stability (8, 9). In the model yeast Saccharomyces cerevisiae, *RAD53* mutants are more sensitive than the wild type to DNA damage and fail to slow cell cycle progression (10); in humans, mutations in *CHK2* are associated with increased breast cancer risk (11).

Despite broad evolutionary conservation in the DNA damage response, recent studies have revealed variation in numerous cell cycle and DNA repair processes among fungi (12–18). For example, the bipolar yeast lineage *Hanseniaspora* has experienced substantial losses among cell cycle and DNA repair genes, which are associated with a punctuated burst of sequence evolution and an increased mutational burden (12). Notwithstanding these losses, *Hanseniaspora* yeasts have successfully diversified and are frequently isolated from the grape and wine must environment (19, 20). Genome instability and hypermutation can also stem from loss of function in a single gene. For example, in a lineage of Cryptococcus deuterogattii yeasts, a nonsense mutation in a DNA mismatch repair gene is associated with an increased mutation rate and rapid evolution of antifungal drug resistance (13, 21).

Beyond fungi, presence and absence patterns among genes responsible for efficacious chromosome segregation also vary across eukaryotes (22). Unraveling how the underlying biological networks compensate for gene losses in living organisms is a challenging task. To overcome this challenge, experimental evolution can be used to provide insight into possible mechanisms that compensate for network perturbation. For example, experimental evolution studies of S. cerevisiae mutants lacking genes responsible for proper chromatid cohesion have revealed distinct evolutionary routes that overcome disrupted chromosome metabolism pathways (23, 24). These findings, together with those in the previous paragraph, support a view that pathways once thought to be resistant to evolutionary change can diverge.

**FIG 1:**
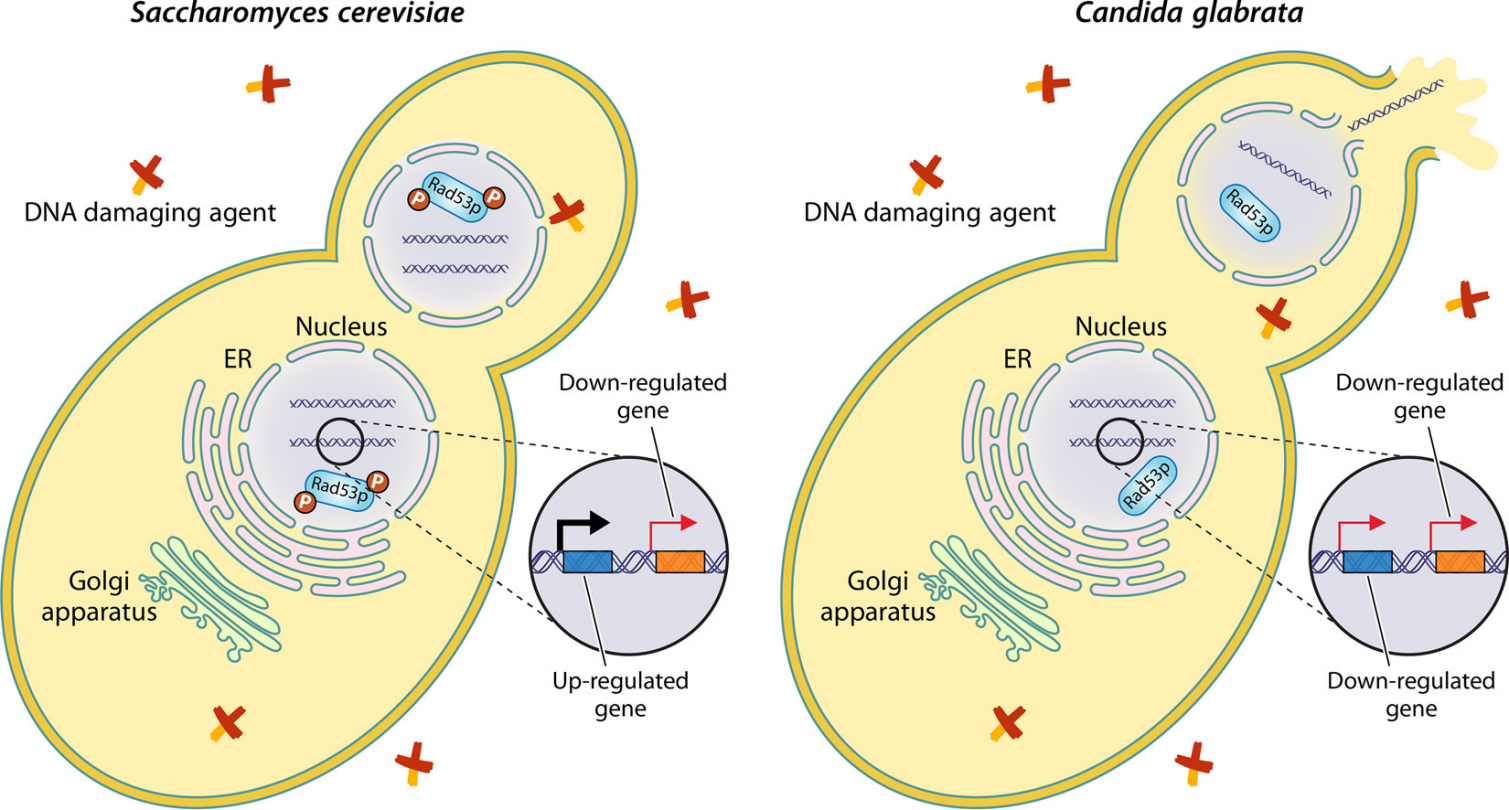
Divergence in the DNA damage response in a model yeast and a major yeast pathogen. (Left) In the model yeast Saccharomyces cerevisiae, the presence of DNA-damaging agents, like methyl methanesulfonate, activates the DNA damage response to help ensure genome integrity. A key step in the DNA damage response is phosphorylation-based activation of Rad53p, which activates multiple downstream processes, including upregulated expression of DNA repair genes, thereby providing the cell with an opportunity to repair DNA damage. (Right) In contrast, the noncanonical DNA damage response in the major yeast pathogen Candida glabrata is marked by reduced Rad53p phosphorylation and is associated with divergent expression of DNA repair genes, which may be responsible for higher rates of mitotic failure and cell death. ER, endoplasmic reticulum.

However, the extent and functional outcome of variation among cell cycle and DNA repair processes remain poorly understood. Furthering our understanding of the diversity and function of the DNA damage response network, Shor et al. (25) characterize a noncanonical DNA damage response in Candida glabrata, a genetically diverse major fungal pathogen comprised of at least seven major lineages (26–28). Of note, in comparison to the more-well-known *Candida* yeast Candida albicans, which is roughly as divergent from S. cerevisiae as humans are from sponges, C. glabrata and S. cerevisiae are more closely related and their divergence is on par with that of humans and zebrafish (29). Shor et al. demonstrate that DNA damage induced less Rad53p phosphorylation in C. glabrata than in S. cerevisiae (Fig. 1), which is likely due to sequence divergence at key phosphorylation sites. In contrast, DNA damage is known to induce robust Rad53p phosphorylation in more distantly related species, including C. albicans (5, 30, 31). These findings suggest that differences likely exist between the DNA damage responses of C. glabrata and S. cerevisiae.

To this end, the researchers examined the DNA damage-induced transcriptome and activities of Rad53p targets in C. glabrata and S. cerevisiae and revealed a divergent architecture, expression, and transcriptional rewiring of the DNA damage response network in C. glabrata (Fig. 1). For example, genes present in the genome of C. glabrata but absent in the genome of S. cerevisiae were frequently upregulated in response to DNA damage. Additionally, genes that encode known targets of Rad53p in S. cerevisiae, such as *RNR3*, which encodes a ribonucleotide reductase subunit, and *HUG1*, which encodes a ribonucleotide reductase inhibitor, are absent from the genome of C. glabrata. Among orthologous genes that contribute to genome integrity, numerous genes were found to be upregulated in S. cerevisiae but downregulated in C. glabrata, including the proliferating cell nuclear antigen (or PCNA, which is encoded by *POL30*), a subunit of the prereplicative complex (*CDC6*), several subunits of the minichromosome maintenance replicative helicase, and a subunit of DNA polymerase δ (*POL31*). Among genome integrity genes that were lost in *Hanseniaspora* yeasts (12), divergent expression profiles were also pronounced, revealing that some genome integrity genes differentially contribute to the DNA damage response networks in diverse yeasts. Despite substantial divergence in the architectures and expression of the DNA damage response networks, some targets of Rad53p that are present in the genomes of both yeasts had similar expression profiles, indicating that their expression was likely mediated through a Rad53p-independent mechanism, a signature of transcriptional rewiring (32).

Downregulation of multiple genes that are responsible for genome integrity suggests that the checkpoint signaling module is less robust in C. glabrata than in S. cerevisiae. Supporting this hypothesis, the researchers reveal that C. glabrata had an attenuated cell cycle checkpoint response in the presence of DNA damage, which may contribute to higher rates of mitotic failure and cell lethality than in S. cerevisiae (Fig. 1). Although hypermutation contributes to pathogenicity-related traits, such as multidrug resistance in diverse fungi, including C. glabrata (13, 21, 33, 34), the contribution of the noncanonical DNA damage response in C. glabrata to pathogenicity and pathogenicity-related phenotypes is unknown but holds promise as an exciting area for future research.

This study, taken together with other recent findings (12, 13, 17, 18, 21, 23, 24), expands our knowledge of the variation among fungal DNA damage response networks. These observations raise the question of what events lead to DNA damage response network perturbation and, in some cases, subsequent stabilization in different lineages. More broadly, these studies contribute to a timely discussion of variation in the architecture, wiring, and function of the eukaryotic DNA damage response.

## References

[1] Friedberg EC, Walker GC, Siede W, Wood RD, Schultz RA, Ellenberger T. 2006. DNA repair and mutagenesis, 2nd ed. ASM Press, Washington, DC.

[2] Giglia-Mari G, Zotter A, Vermeulen W. 2011. DNA damage response. Cold Spring Harb Perspect Biol 3:a000745. doi:10.1101/cshperspect.a000745. 20980439 PMC3003462

[3] Barnum KJ, O’Connell MJ. 2014. Cell cycle regulation by checkpoints. Methods Mol Biol 1170:29–40. doi:10.1007/978-1-4939-0888-2_2. 24906307 PMC4990352

[4] D’Andrea AD. 2014. DNA repair pathways and human cancer. The molecular basis of cancer, 4th ed. Elsevier Inc., Philadelphia, PA.

[5] Jung K-W, Lee Y, Huh EY, Lee SC, Lim S, Bahn Y-S. 2019. Rad53- and Chk1-dependent DNA damage response pathways cooperatively promote fungal pathogenesis and modulate antifungal drug susceptibility. mBio 10:e01726-18. doi:10.1128/mBio.01726-18. 30602579 PMC6315099

[6] Ma J-L, Lee S-J, Duong JK, Stern DF. 2006. Activation of the checkpoint kinase Rad53 by the phosphatidyl inositol kinase-like kinase Mec1. J Biol Chem 281:3954–3963. doi:10.1074/jbc.M507508200. 16365046

[7] Sanchez Y, Desany BA, Jones WJ, Liu Q, Wang B, Elledge SJ. 1996. Regulation of RAD53 by the ATM-like kinases MEC1 and TEL1 in yeast cell cycle checkpoint pathways. Science 271:357–360. doi:10.1126/science.271.5247.357. 8553072

[8] Pellicioli A, Foiani M. 2005. Signal transduction: how Rad53 kinase is activated. Curr Biol 15:R769–R771. doi:10.1016/j.cub.2005.08.057. 16169479

[9] Elledge SJ. 1996. Cell cycle checkpoints: preventing an identity crisis. Science 274:1664–1672. doi:10.1126/science.274.5293.1664. 8939848

[10] Paulovich AG, Hartwell LH. 1995. A checkpoint regulates the rate of progression through S phase in S. cerevisiae in response to DNA damage. Cell 82:841–847. doi:10.1016/0092-8674(95)90481-6. 7671311

[11] Apostolou P, Papasotiriou I. 2017. Current perspectives on CHEK2 mutations in breast cancer. Breast Cancer (Dove Med Press) 9:331–335. doi:10.2147/BCTT.S111394. 28553140 PMC5439543

[12] Steenwyk JL, Opulente DA, Kominek J, Shen X-X, Zhou X, Labella AL, Bradley NP, Eichman BF, Čadež N, Libkind D, DeVirgilio J, Hulfachor AB, Kurtzman CP, Hittinger CT, Rokas A. 2019. Extensive loss of cell-cycle and DNA repair genes in an ancient lineage of bipolar budding yeasts. PLoS Biol 17:e3000255. doi:10.1371/journal.pbio.3000255. 31112549 PMC6528967

[13] Billmyre RB, Clancey SA, Heitman J. 2017. Natural mismatch repair mutations mediate phenotypic diversity and drug resistance in Cryptococcus deuterogattii. Elife 6:e28802. doi:10.7554/eLife.28802. 28948913 PMC5614558

[14] Rhodes J, Beale MA, Vanhove M, Jarvis JN, Kannambath S, Simpson JA, Ryan A, Meintjes G, Harrison TS, Fisher MC, Bicanic T. 2017. A population genomics approach to assessing the genetic basis of within-host microevolution underlying recurrent cryptococcal meningitis infection. G3 (Bethesda) 7:1165–1176. doi:10.1534/g3.116.037499. 28188180 PMC5386865

[15] Boyce KJ, Wang Y, Verma S, Shakya VPS, Xue C, Idnurm A. 2017. Mismatch repair of DNA replication errors contributes to microevolution in the pathogenic fungus Cryptococcus neoformans. mBio 8:e00595-17. doi:10.1128/mBio.00595-17. 28559486 PMC5449657

[16] Mitchison-Field LMY, Vargas-Muñiz JM, Stormo BM, Vogt EJD, Van Dierdonck S, Pelletier JF, Ehrlich C, Lew DJ, Field CM, Gladfelter AS. 2019. Unconventional cell division cycles from marine-derived yeasts. Curr Biol 29:3439–3456.e5. doi:10.1016/j.cub.2019.08.050. 31607535 PMC7076734

[17] Milo S, Misgav RH, Hazkani-Covo E, Covo S. 2019. Limited DNA repair gene repertoire in ascomycete yeast revealed by comparative genomics. Genome Biol Evol 11:3409–3423. doi:10.1093/gbe/evz242. 31693105 PMC7145719

[18] Cohen R, Milo S, Sharma S, Savidor A, Covo S. 2019. Ribonucleotide reductase from Fusarium oxysporum does not respond to DNA replication stress. DNA Repair (Amst) 83:102674. doi:10.1016/j.dnarep.2019.102674. 31375409

[19] Martin V, Valera M, Medina K, Boido E, Carrau F. 2018. Oenological impact of the Hanseniaspora/Kloeckera yeast genus on wines—a review. Fermentation 4:76. doi:10.3390/fermentation4030076.

[20] Chavan P, Mane S, Kulkarni G, Shaikh S, Ghormade V, Nerkar DP, Shouche Y, Deshpande MV. 2009. Natural yeast flora of different varieties of grapes used for wine making in India. Food Microbiol 26:801–808. doi:10.1016/j.fm.2009.05.005. 19835764

[21] Billmyre RB, Applen Clancey S, Li LX, Doering TL, Heitman J. 2020. 5-Fluorocytosine resistance is associated with hypermutation and alterations in capsule biosynthesis in Cryptococcus. Nat Commun 11:127. doi:10.1038/s41467-019-13890-z. 31913284 PMC6949227

[22] Kops GJPL, Snel B, Tromer EC. 2020. Evolutionary dynamics of the spindle assembly checkpoint in eukaryotes. Curr Biol 30:R589–R602. doi:10.1016/j.cub.2020.02.021. 32428500

[23] Hsieh Y-YP, Makrantoni V, Robertson D, Marston AL, Murray AW. 2020. Evolutionary repair: changes in multiple functional modules allow meiotic cohesin to support mitosis. PLoS Biol 18:e3000635. doi:10.1371/journal.pbio.3000635. 32155147 PMC7138332

[24] Fumasoni M, Murray AW. 2020. The evolutionary plasticity of chromosome metabolism allows adaptation to constitutive DNA replication stress. Elife 9:e51963. doi:10.7554/eLife.51963. 32043971 PMC7069727

[25] Shor E, Garcia-Rubio R, DeGregorio L, Perlin DS. 2020. A noncanonical DNA damage checkpoint response in a major fungal pathogen. mBio 11:e03044-20. doi:10.1128/mBio.03044-20. 33323516 PMC7773997

[26] Carreté L, Ksiezopolska E, Pegueroles C, Gómez-Molero E, Saus E, Iraola-Guzmán S, Loska D, Bader O, Fairhead C, Gabaldón T. 2018. Patterns of genomic variation in the opportunistic pathogen Candida glabrata suggest the existence of mating and a secondary association with humans. Curr Biol 28:15–27.e7. doi:10.1016/j.cub.2017.11.027. 29249661 PMC5772174

[27] Muller H, Thierry A, Coppée J-Y, Gouyette C, Hennequin C, Sismeiro O, Talla E, Dujon B, Fairhead C. 2009. Genomic polymorphism in the population of Candida glabrata: gene copy-number variation and chromosomal translocations. Fungal Genet Biol 46:264–276. doi:10.1016/j.fgb.2008.11.006. 19084610

[28] Carreté L, Ksiezopolska E, Gómez-Molero E, Angoulvant A, Bader O, Fairhead C, Gabaldón T. 2019. Genome comparisons of Candida glabrata serial clinical isolates reveal patterns of genetic variation in infecting clonal populations. Front Microbiol 10:112. doi:10.3389/fmicb.2019.00112. 30809200 PMC6379656

[29] Shen X-X, Opulente DA, Kominek J, Zhou X, Steenwyk JL, Buh KV, Haase MAB, Wisecaver JH, Wang M, Doering DT, Boudouris JT, Schneider RM, Langdon QK, Ohkuma M, Endoh R, Takashima M, Manabe R, Čadež N, Libkind D, Rosa CA, DeVirgilio J, Hulfachor AB, Groenewald M, Kurtzman CP, Hittinger CT, Rokas A. 2018. Tempo and mode of genome evolution in the budding yeast subphylum. Cell 175:1533–1545.e20. doi:10.1016/j.cell.2018.10.023. 30415838 PMC6291210

[30] Kapitzky L, Beltrao P, Berens TJ, Gassner N, Zhou C, Wüster A, Wu J, Babu MM, Elledge SJ, Toczyski D, Lokey RS, Krogan NJ. 2010. Cross-species chemogenomic profiling reveals evolutionarily conserved drug mode of action. Mol Syst Biol 6:451. doi:10.1038/msb.2010.107. 21179023 PMC3018166

[31] Wang H, Gao J, Li W, Wong AH-H, Hu K, Chen K, Wang Y, Sang J. 2012. Pph3 dephosphorylation of Rad53 is required for cell recovery from MMS-induced DNA damage in Candida albicans. PLoS One 7:e37246. doi:10.1371/journal.pone.0037246. 22606354 PMC3351423

[32] Dalal CK, Zuleta IA, Mitchell KF, Andes DR, El-Samad H, Johnson AD. 2016. Transcriptional rewiring over evolutionary timescales changes quantitative and qualitative properties of gene expression. Elife 5:e18981. doi:10.7554/eLife.18981. 27614020 PMC5067116

[33] Healey KR, Jimenez Ortigosa C, Shor E, Perlin DS. 2016. Genetic drivers of multidrug resistance in Candida glabrata. Front Microbiol 7:1995. doi:10.3389/fmicb.2016.01995. 28018323 PMC5156712

[34] Dellière S, Healey K, Gits-Muselli M, Carrara B, Barbaro A, Guigue N, Lecefel C, Touratier S, Desnos-Ollivier M, Perlin DS, Bretagne S, Alanio A. 2016. Fluconazole and echinocandin resistance of Candida glabrata correlates better with antifungal drug exposure rather than with MSH2 mutator genotype in a French cohort of patients harboring low rates of resistance. Front Microbiol 7:2038. doi:10.3389/fmicb.2016.02038. 28066361 PMC5179511

